# Legacies of Lead in Charm City’s Soil: Lessons from the Baltimore Ecosystem Study

**DOI:** 10.3390/ijerph13020209

**Published:** 2016-02-06

**Authors:** Kirsten Schwarz, Richard V. Pouyat, Ian Yesilonis

**Affiliations:** 1 Department of Biological Sciences, Northern Kentucky University, Nunn Drive, Highland Heights, KY 41099, USA; 2 USDA Forest Service, Research & Development, Washington, DC 20250, USA; rpouyat@fs.fed.us; 3 USDA Forest Service, 5523 Research Park, Suite 350, Baltimore, MD 21228, USA; iyesilonis@fs.fed.us

**Keywords:** urban, soil, Baltimore Ecosystem Study, lead, heavy metals

## Abstract

Understanding the spatial distribution of soil lead has been a focus of the Baltimore Ecosystem Study since its inception in 1997. Through multiple research projects that span spatial scales and use different methodologies, three overarching patterns have been identified: (1) soil lead concentrations often exceed state and federal regulatory limits; (2) the variability of soil lead concentrations is high; and (3) despite multiple sources and the highly heterogeneous and patchy nature of soil lead, discernable patterns do exist. Specifically, housing age, the distance to built structures, and the distance to a major roadway are strong predictors of soil lead concentrations. Understanding what drives the spatial distribution of soil lead can inform the transition of underutilized urban space into gardens and other desirable land uses while protecting human health. A framework for management is proposed that considers three factors: (1) the level of contamination; (2) the desired land use; and (3) the community’s preference in implementing the desired land use. The goal of the framework is to promote dialogue and resultant policy changes that support consistent and clear regulatory guidelines for soil lead, without which urban communities will continue to be subject to the potential for lead exposure.

## 1. Introduction

The legacy of lead in Baltimore’s soil has been a topic of environmental and social inquiry for decades. The Baltimore Ecosystem Study (BES)—a long-term ecological research project funded by the National Science Foundation—has contributed to this line of inquiry using multiple methods that cross spatial and temporal scales [1,2,3]. The central aim of this paper is to draw conclusions from that body of research that can inform policy and advance solutions for sustainable and livable cities.

Lead is naturally found in soils at very low concentrations—a median of 11 ppm for US agricultural soils [4]. However, lead has entered soil systems through the historic combustion of leaded gasoline and the deterioration of lead-based paint [5], as well as multiple industrial sources, including smelters [6], incinerators [7], and coal-burning plants [8]. While consumer products such as gasoline and paint no longer contain lead, their past use has resulted in the accumulation of lead in the environment, with four to five million metric tons deposited from leaded-fuel alone [9]. In addition to the widespread extent of soil contamination, lead enriched soil is mobile, with the potential to be redistributed in the environment when soil particles move with wind and water [10].

Soil contaminated with lead has direct human health consequences, becoming a source to human populations when it is inhaled or ingested—even very small amounts of lead in the body (<10 µg/dL) have been linked to adverse health effects [11,12,13,14]. Children are especially vulnerable to lead exposure because of their high rates of hand-to mouth activity [15] and developing bodies. In addition, household dust and educational interventions are not effective at reducing children’s blood lead levels [16] highlighting the need to expand lead prevention efforts to include soil systems [5,12,17].

While soil systems can be managed for soil lead through amendments that reduce bioavailability [18], removing contaminated soil, or creating barriers between soil and humans, the success of any intervention depends on identifying areas of elevated soil lead, sometimes referred to as “hotspots.” Findings from the BES are summarized to shed light on the spatial distribution of lead in urban social-ecological systems and suggest a framework for evaluating and implementing soil lead remediation plans in urban communities. The application of these lessons is especially relevant to communities as cities shift from “sanitary” to “sustainable” [19], transforming underutilized urban space to green infrastructure. This transformation often includes the expansion or creation of urban gardens in which tradeoffs may occur between gardening and human exposure to soil contaminated by lead [20,21,22,23].

## 2. Methods

Within the last decade, studies from the BES have advanced the understanding of both the amount and spatial distribution of lead in urban soil and identified important drivers of soil lead concentrations. The goal of this paper is to summarize findings from these studies, derived from observations at the city, neighborhood, and parcel scales [1,2,3]. Combined, these data address soil lead loadings from both local point sources such as lead-based paint, and local and regional non-point sources such as leaded fuel and atmospheric deposition.

*City Scale*—As part of the BES, 125 study plots were established in 1999 to calibrate the Urban Forest Effects Model, developed by the United States Department of Agriculture Forest Service, Northeastern Research Center to characterize the structure of urban forests [24]. Plots span Baltimore City and were selected using a stratified random sampling scheme—stratified by land use using Anderson Level II land cover classes [25] and weighted by area (see Pouyat *et al.* [1] for more details on study design). Composite soil samples were collected at each plot from the surface 10 cm in the summer of 2000. Samples were air dried, passed through a 2-mm sieve and acid digested using a modified USEPA method 3050B [26] at the BES and University of Maryland, Baltimore County lab. Samples were analyzed for lead by Inductively Coupled Plasma at the Cornell University Nutrient Analysis Lab [1]. At the city scale, multivariate and *post-hoc* univariate statistical analyses were conducted to test whether soil properties differed among land use/cover and surface geology. Using the same plot data, the spatial distribution of soil lead concentrations was investigated.

*Neighborhood Scale*—Surface soil metals and nutrients were measured in Watershed 263, a 376 ha storm sewer watershed in Baltimore City’s Harbor Watershed that outfalls to the Middle Branch. This watershed area is home to 30,000 residents and is entirely urbanized with mixed industrial, institutional, and residential land uses, as well as significant public parkland and private parkland. Composite samples (0–5 cm) were collected from 39 plots that were stratified by a land use/cover classification system [27]. Samples were air dried, ground and passed through a 2-mm sieve. Samples were digested at the BES and University of Maryland Baltimore County Lab using a nitric extraction technique. The extracted samples were sent to the Cornell University Nutrient Analysis Laboratory to determine acid soluble concentrations of lead using an Inductively Coupled Plasma Atomic Emission Spectrophotometer.

*Parcel Scale*—Sixty-one owner-occupied residential properties were intensively sampled for soil lead in Baltimore City in 2007 using a Niton XLt 700 series handheld X-ray fluorescence multiple element analyzer [2]. The central goal of the research was to identify the fine scale heterogeneity of soil lead in residential areas. Parcels were selected using a stratified sampling scheme—stratified by (1) housing age (1986-2007 and pre-1978) to account for the 1978 ban on lead-based paint and the 1986 ban on leaded gasoline; (2) distance to major road networks (0–30 m, 30+ m) to account for historic leaded gasoline deposition; and (3) housing material (brick and wood frame) to account for the likelihood of painted surfaces. Soil lead content to a depth of approximately 2 mm was evaluated *in situ*—a minimum of 5% of *in situ* samples were confirmed via laboratory analyses at an independent USEPA recognized lab using Atomic Absorption Spectroscopy analyses [2].

## 3. Lessons Learned

Three main lessons learned from observations of soil lead in Baltimore City have been identified: (1) soil lead concentrations often exceeded state and federal regulatory limits; (2) the variability of soil lead concentrations was high at all scales of observation; and (3) despite multiple sources and the highly heterogeneous and patchy nature of soil lead, discernable patterns existed at all scales analyzed.

### 3.1. Lesson 1—Soil Lead Exceeds Regulatory Limits

There are relatively large differences among surface soil guidelines for heavy metal contamination both in North America [28] and globally [29]. In a worldwide analysis of surface soil guidelines, soil lead guidelines were found to span 3.7 orders of magnitude [30]. Within the United States, there are multiple regulatory limits among state and federal agencies. The US Environmental Protection Agency has two soil lead guidelines: 400 ppm for children’s play areas and 1200 ppm for all other areas of the yard. The US national standard of 400 ppm is high compared to standards used in other countries [30]. The geometric mean of regulatory guidance values for the US is 268 ppm, while it is 167 ppm elsewhere in the world [30]. Some states in the US have adopted more stringent guidelines compared to the US national standard of 400 ppm. For example, the CA Office of Environmental Health Hazard Assessment uses a guideline of 80 ppm. Guidelines have also been considered in the context of specific land uses—a recent working group from the US EPA, tasked with examining soil lead guidelines for urban gardening activities, recommended a guideline of 100 ppm [31]. For the purposes of this paper, results are compared to the US national standard of 400 ppm as well as a more conservative guideline, specifically the CA Office of Environmental Health Hazard Assessment guideline of 80 ppm.

Studies from Baltimore, MD that span spatial scales and use different methodologies reached the same conclusion—soil lead values often exceeded the US national standard of 400 ppm. This supports pioneering research in Baltimore, MD conducted more than three decades ago that first demonstrated that lead has accumulated in urban soils at levels that can produce negative human health outcomes [17].

**Figure 1:**
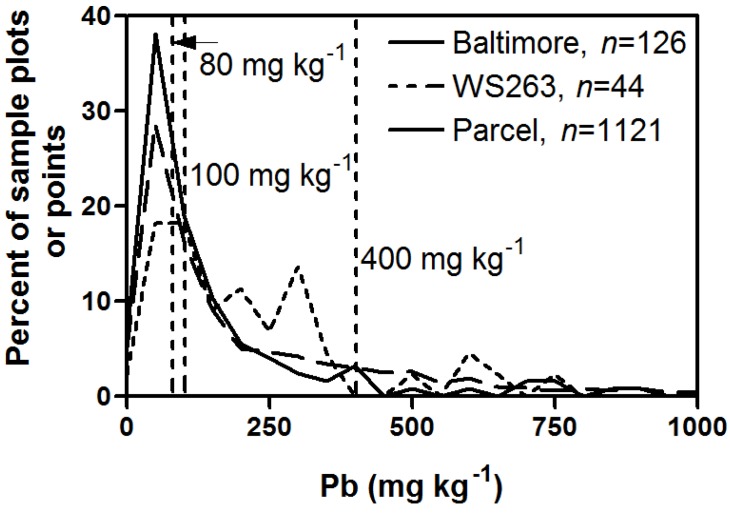
Plot frequency distributions of the percentage of sample plots or points having a specific lead concentration (mg·kg^−1^) measured for soils in the Watershed 263 neighborhood (solid line, plots), Baltimore City (dashed line, plots), and Baltimore City parcel (thin dashed line, points). A plot is a 0.04-hectare area where a composite sample was collected and analyzed for lead and a point is a lead value determined by X-ray fluorescence multiple element analyzer on a residential property. There was one value for a plot and multiple for a residential property. The U.S. Environmental Protection Agency (EPA) soil screening levels (400 mg·kg^−1^), the EPA Technical Review Workgroup for Lead recommendation for urban gardening (100 mg·kg^−1^), and California state soil guideline (80 mg·kg^−1^) values are also shown. Data sources: Baltimore [3], Watershed 263 (unpublished), and Parcel [2].

At a citywide scale, soil lead concentrations exceeded the US national guideline of 400 ppm and the CA soil guideline of 80 ppm in 10% and 55% of sampled soils, respectively (Figure 1, Table 1). Similarly, but with up to 60% more occurrences, soil lead concentrations in an older and economically depressed neighborhood in southwest Baltimore (Watershed 263) exceeded the US national guideline of 400 ppm and the CA soil guideline of 80 ppm in 16% and 77% of sampled soils, respectively (Figure 1, Table 1). At the parcel scale, 22% of soil samples exceeded 400 ppm, while 63% of soil samples exceeded 80 ppm (Figure 1, Table 1). The parcel scale data were used to develop several predictive models for Baltimore, MD using three different approaches—a general linear model, classification and regression trees, and Random Forest [32]. The models predict varying amounts of soil lead in excess of 400 ppm—ranging from 6% to 29% of the total modeled area [32].

**Table 1:** Percentage of plots (Watershed 263 and Baltimore City) or points (Parcel) that exceed the lead guideline values of 80, 100, 400, and 1200 mg·kg^−1^ and summary statistics. A plot is a 0.04-hectare area where a composite sample was collected and analyzed for lead and a point is a lead value determined by an X-ray fluorescence multiple element analyzer on a residential property. There was one value for a plot and multiple for a residential property. Data sources: Baltimore [3], Watershed 263 (unpublished), and Parcel [2].

Guideline Value (mg·kg^−1^)	80	100	400	1200	Min. Pb (ppm)	Max. Pb (ppm)	Mean Pb (ppm)	Standard Deviation	*n*
Parcel	63**%**	56**%**	22**%**	6**%**	7	9151	363	794	1121
Watershed 263	77**%**	73**%**	16**%**	2**%**	22	2495	292	405	43
Baltimore City	55**%**	43**%**	10**%**	2**%**	4	5652	229	572	126

Many studies have demonstrated that urban soils are elevated in lead and other metals. The combined results of BES demonstrate that soil lead patterns are consistently influenced by only a few drivers operating at multiple scales. The city and neighborhood scale composite samples show widespread contamination across the city primarily related to roads and age of structure, while the parcel measurements show contamination at much higher levels because the sampling was done at a much finer resolution allowing for quantification of soil lead closer to suspected sources. While some of the difference can be attributed to differences in data collection, not all of the variation can be explained by methodology alone. Soil lead patterns are a combination of elevated urban background contamination and local hotspots—both of which have implications for land use management.

### 3.2. Lesson 2—The Variability of Soil Lead Is High

Given the exceedances found in observations from Baltimore and results from many other cities, soil lead is greatly elevated in urban areas, well beyond background levels. Although soil lead concentrations are elevated, the distribution is uneven introducing a high amount of variability in the data (Table 1). The high variability of soil lead has important implications for land-use planning as well as sampling designs. Knowledge regarding the distribution of highly contaminated areas, or “hotspots” at multiple scales, is necessary to inform the placement of gardens, play areas, and other land uses where humans and the soil systems closely interact.

**Figure 2:**
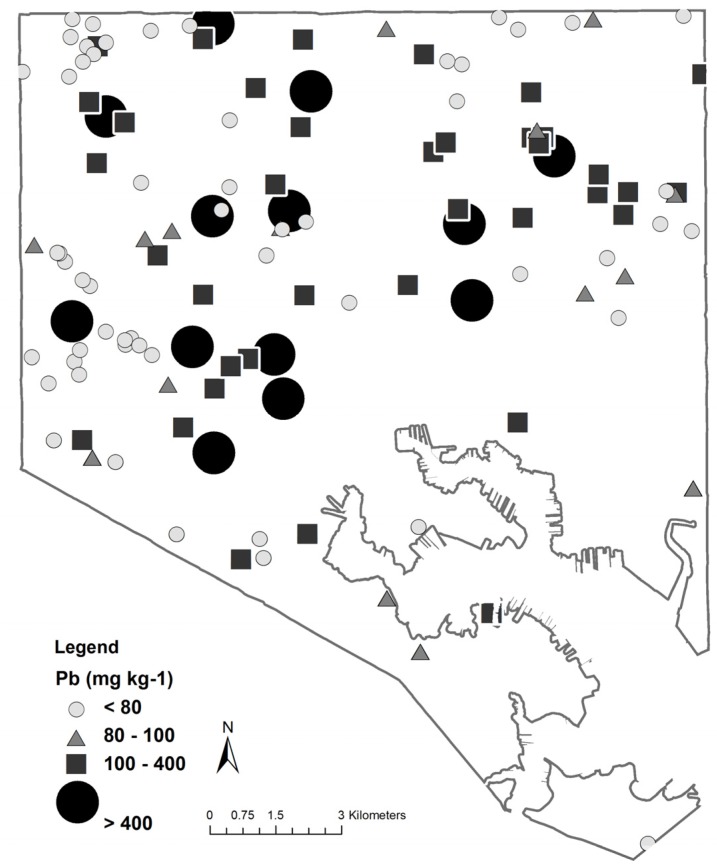
Map showing the spatial distribution of lead concentration (mg·kg^−1^) within Baltimore City, MD, USA. The U.S. Environmental Protection Agency (EPA) soil screening levels (400 mg·kg^−1^), the EPA Technical Review Workgroup for Lead recommendation for urban gardening (100 mg·kg^−1^), and California state soil guideline (80 mg·kg^−1^) values are represented in the legend. Data from [3].

The high variability of soil lead is inextricably linked to the spatial heterogeneity of soil lead and patterns found on the landscape. This is due to multiple drivers working at several different spatial scales. A citywide map showing the spatial distribution of lead concentrations confirms that contamination is widespread and variable (Figure 2). The patterns produced by the high amount of variability are in part due to factors specific to urban landscapes, which are highlighted below.

### 3.3. Lesson 3—Patterns Exist

While the variability is high and the extent of contamination is wide, discernable patterns do exist for soil lead levels at multiple scales, which makes predicting potential hotspots and the management of soil lead in urban landscapes possible. Prediction cannot replace soil testing, but understanding the drivers of soil lead distribution can assist in prioritizing sampling and remediation efforts, inform urban design, and identify areas where changing the relationship between humans and soil systems would have the largest impact.

Lead contamination in urban soils has been associated with roadside environments and vehicular emissions [33,34,35,36], interior and exterior paint [37], stack emissions [38,39,40], and management inputs [41]. These sources and their dispersal at multiple scales have been shown to result in predictable patterns in several metropolitan areas in the United States and Europe. For example, in the Baltimore, New York City, and Budapest metropolitan areas, Pouyat *et al.* [42] found soil lead loadings in forest fragments along an urban-rural gradient to be highly correlated with distance from the city core, percent urban land, traffic volume, and road density. A similar pattern but with greater differences was found more than three decades ago by Inman and Parker [43] in the Chicago, IL metropolitan area, where levels of lead were more than five times higher in urban than in rural forest patches. Other urbanization gradient studies have shown a similar pattern [44], although smaller cities or cities having more compact development patterns exhibited less of a difference between urban and rural forest soils [42,45,46].

Using a similar study design but for residential yards, Pouyat *et al.* [36] compared soil lead concentrations in the Baltimore-Washington metropolitan area and found that urban yards exhibited up to 10-fold higher concentrations than in rural yards. Moreover, these differences were greater for older parcels and structures. For residential yards and urban soils in the United States, a recent review of the literature showed that for all studies considered, soils in urban centers had higher lead concentrations than in suburban areas, with the exception of New Orleans [47]. By contrast to most metropolitan areas in the United States, residential yards did not exhibit high levels of lead when compared to other land use types in Beijing, China [48].

**Figure 3:**
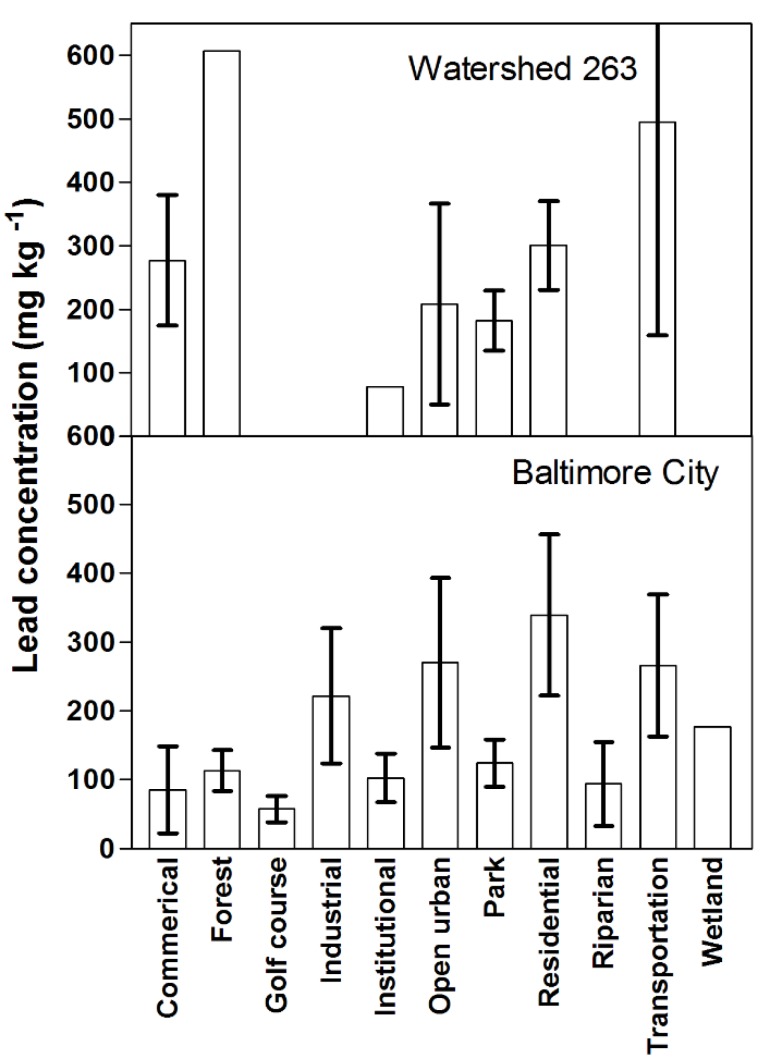
Mean (including standard error) concentrations of soil lead (0–10 cm depth) by land use and cover type for Watershed 263 and Baltimore City. Data sources: Baltimore [1], Watershed 263 (unpublished).

The contamination of soil by lead in natural fragments of vegetation and in residential yards and its direct relationship to various measures of urban land uses, in particular vehicle use along urbanization gradients, suggests a dispersion pattern from the edge of a road in tens to hundreds of meters [42]. Additionally, the contamination is related to vehicle usage and the length of time the contamination has occurred—the more traffic and the older the road the greater the contamination—which is true for both forest fragments and residential yards. Taking these observations from the metropolitan to city scale, Pouyat *et al.* [1] found lead concentrations were relatively high in residential areas and surprisingly did not statistically differ from the institutional and commercial/industrial areas sampled in Baltimore City (Figure 3). Later research in Baltimore confirmed that land use was not a useful predictor of soil lead concentrations [49]. Additionally, within the residential areas, the age of structure was related to soil lead with structures built prior to 1930 having up to a 10-fold higher concentrations than structures built after 1930 [3]. Also at the city scale, Yesilonis *et al.* [3] found a relationship between roads and lead and zinc soil concentrations. Levels of these trace metals with respect to major roads in Baltimore were 3.5- and 2.8-fold higher inside than outside a 100-m buffer zone for lead and zinc, respectively. Both of these trace metals can be associated with vehicle use.

Soil lead data have also been collected at the higher resolution of a neighborhood (approximately 376 ha) in Baltimore City (Watershed 263). Watershed 263 is a storm sewer watershed in southwest Baltimore and is predominantly made up of high-density residential areas with all of the sampling plots falling within the 100-meter buffer that was reported in Yesilonis *et al.* (2008). Similar to the citywide results, lead concentrations were not significantly explained by land use and cover. Land use associated with transportation was the highest and most variable with an average soil lead concentration of 500 ppm compared to 300 ppm or lower for the other land use types (Figure 3). In addition, means for all of the land use and cover types were higher than the CA soil lead guideline of 80 ppm.

X-ray fluorescence (XRF) field measurements at the parcel scale showed that the variability of soil lead is very high—sometimes differing by an order of magnitude over a few meters [2], which is consistent with measurements at coarser scales (Table 1). Regardless of the high variability, the Schwarz *et al.* [2] observations show that housing age at the parcel scale continued as an important predictor of soil lead. Similar to the city scale, the relationship with age at the parcel scale can be interpreted as the likelihood that lead-based paints were used and the amount of time that a structure has been intercepting atmospheric deposition [2]. In addition, lead levels are often elevated on built surfaces facing major roadways [2,32].

**Figure 4:**
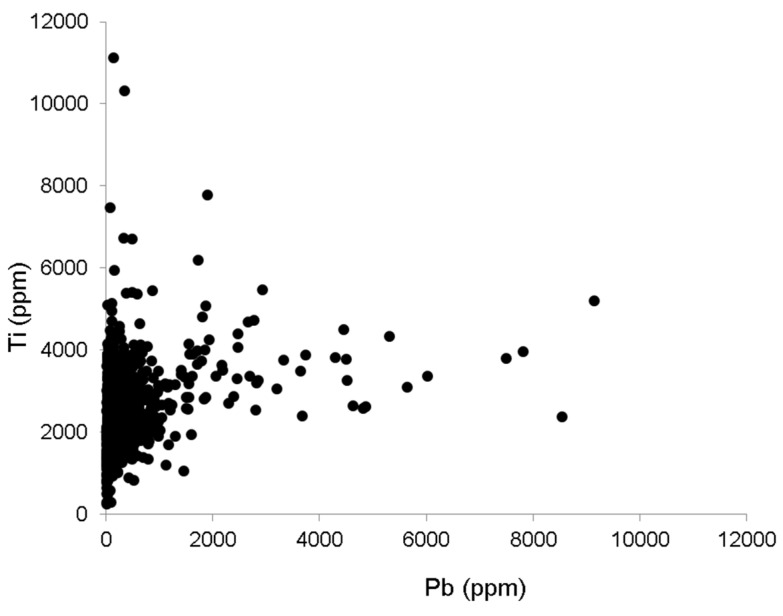
Lead and titanium concentrations do not show a strong relationship. If paint were the only source of lead to soil, a stronger correlation between the two elements would be expected. Data source: [49].

**Figure 5:**
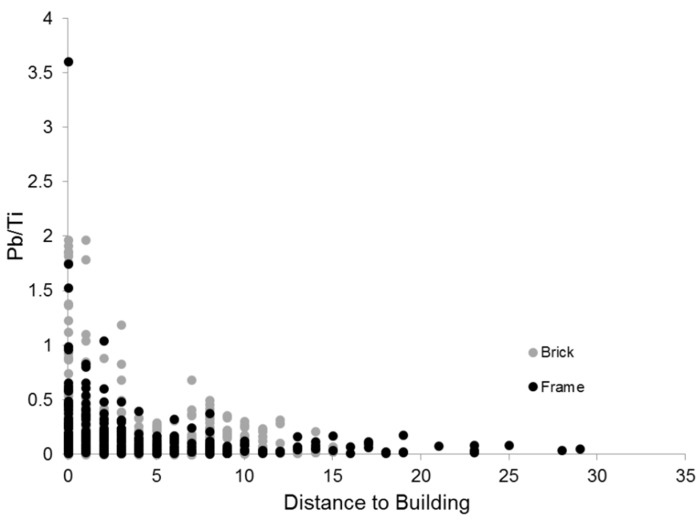
Lead/titanium ratios and distance to building. Ratios are higher close to the building and decrease with distance. Ratios greater than one highlight the possibility of multiple sources of lead in the soil. Date source: [49].

The overall results from studies of urban soil lead in Baltimore suggest two spatial patterns associated with lead: (1) there are multiple sources of lead (leaded-gasoline and lead-based paint); and (2) both sources have contributed to widespread soil lead pollution in Baltimore even after more than two decades of restrictions on both sources. Element ratios, specifically lead/titanium have been used to determine the source of lead in soil [20]. Titanium is a component of both lead-based paint (lead titanate) and lead-free paint (titanium dioxide) and therefore an indicator of total paint products in soil. Soils contaminated with lead-based paint exhibit enrichment of both lead and titanium, while soils contaminated with leaded-gasoline exhibit higher lead levels compared to titanium [20]. In Baltimore, lead and titanium do not appear to be enriched in the same way, as evidenced by the lack of a strong correlation between the two elements using the parcel data (*p* ≤ 0.0001, *r*^2^ = 0.067, Figure 4). If paint were the only source of lead to soil, one would expect a stronger correlation between the two elements. In addition, other studies have shown that lead/titanium ratios from non-urban paint sources are typically less than 1 [20]. Samples collected at the parcel scale exhibited lead/titanium ratios greater than 1 for samples collected next to both brick and wood frame buildings indicating titanium concentrations are lower than lead concentrations (Figure 5). This suggests multiples sources of lead, such as lead-based paint and leaded gasoline, contribute to soil lead loadings.

## 4. A Framework for Management

The lessons learned from the BES contribute to the growing body of literature on urban soil lead patterns and identify important drivers of soil lead contamination in urban areas. Lead contamination in older cities is pervasive, including residential areas, and thus is an important public health concern that threatens vulnerable populations. Data from the BES can help soil scientists, managers, policy makers, and residents better understand and predict patterns of soil lead and the potential for lead contamination from the city to the parcel scale. Baltimore represents a complex landscape where soil lead concentrations are elevated, variable, and stem from several sources, and yet overarching patterns are predictable at multiple spatial scales.

**Figure 6:**
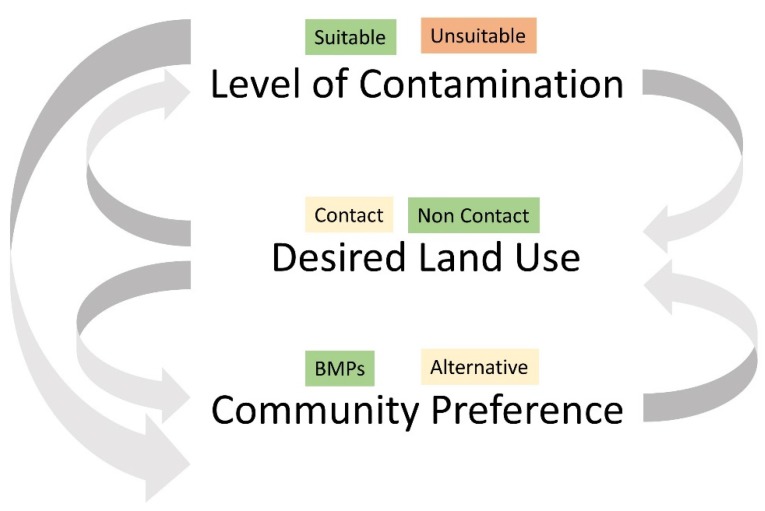
A framework for soil lead management. The framework considers: (1) the level of contamination; (2) the desired land use; and (3) the community’s preference in implementing that desired land use, and interactions among the variables. **Level of contamination** includes suitable and unsuitable, a distinction that the framework does not define, yet highlights the need for consistent soil lead guidelines to inform this decision. **Desired land use** is divided into contact (e.g., gardening and children’s play areas) and non-contact (e.g., landscaped) categories, which determine the amount of interaction between humans and soil systems. **Community preference** is divided into best management practices (BMPs) and alternatives.

While understanding the spatial patterning and variability of soil lead in urban systems can inform and guide management, there are limitations to spatial modeling that should be considered. Work in Baltimore suggests that less than half the variability of lead concentrations can be explained by spatial analyses at multiple scales of observation. With its focus on biogeophysical systems, soil lead research conducted as part of the BES can assist communities and municipalities to predict where soil lead may be elevated, which in turn can determine the need for sampling and help inform remediation efforts. However, in isolation, what has been learned from research in Baltimore does little to advance the successful management of urban soil systems—the lessons learned from Baltimore must be considered within the larger societal and ecological context. A framework for management is presented as a way to stimulate policies that provide clearer guidelines that are both protective of human health and supportive of urban transformations. The framework considers: (1) the level of contamination; (2) the desired land use; and (3) the community’s preference in implementing that desired land use (Figure 6).

**Level of contamination**—Since the 1980s, scientists have brought attention to the contribution of soil lead loadings to children’s blood lead levels [17]. More recent work has empirically established that soil lead loadings can contribute to elevated blood lead levels in children [50]. An important caveat to note is that total soil lead values do not necessarily directly translate to public health concerns. For example, total lead is limited in its ability to predict bioavailability [51]. In addition, the relationship between soil lead and human health risk is complicated by several factors including soil type, pH, soil organic matter, particle size, and lead species—all which can affect bioavailability [52]. However, knowing the lead concentration of the soil is an important step, given the higher the concentration of lead in soil, the greater the risk for exposure. Therefore the level of contamination is a variable to consider in the framework. This information can dictate whether it is safe to implement the desired land use and may result in a new outcome depending on the level of contamination.

At high levels of lead contamination, urban soil can pose a risk to human health; however, the threshold that determines what is safe and what is not continues to be debated [11]. Conflicting soil lead guidelines—both federal and state—create confusion and challenge effective communication of human health risk and scientific uncertainty. For example, based on the analysis, exceedances at all spatial scales vary significantly depending on the guideline used (Table 1). The intention with this paper is not to contribute to that debate—either by identifying the most appropriate threshold or by proposing a new one—the framework simply recognizes that an important step in urban soil transformations is determining whether or not the soil is suitable for human activity and clearly communicating the associated risk with the public. Without this fundamental knowledge, decisions made using any framework are less effective. Consistent guidelines that determine suitability are essential to informed and successful urban transformations.

**Desired land use**—While the level of contamination may influence the desired land use, the desired land use may prompt communities to test for the amount of lead. How communities plan to use soil systems has very important implications for exposure and therefore management. The framework divides land use into two broad categories: “contact” and “non-contact.” Some activities, such as gardening and recreational fields, place humans in close proximity to soil systems, which can become sources of lead to humans when soil is ingested or inhaled. The framework considers these land uses as “contact” land uses. In “contact” land uses, the thresholds for soil contamination would be lower and modulating factors affecting its human health risk would be more relevant to understand. Other uses such as well-established lawns, landscaping, and pavers that overlie soil provide a barrier between humans and soil and may therefore be considered “non-contact.” For the purposes of the framework, “contact” land use should be treated with more caution than “non-contact” land use because of the greater likelihood of exposure. “Non-contact” land use could be contaminated up to the level of contamination determined suitable for human activity, where “contact” land use would require a lower threshold. It is important, however, to recognize that “non-contact” land use can change over time, becoming a source to human populations. For example, bare spots can develop in a lawn and become sources of lead, especially in dry and windy conditions.

**Community preference**—Just as the desired land use can influence the community’s preference in implementing that land use, the community’s preference can also have important feedbacks to the desired land use. Full disclosure of the level and distribution of soil lead is essential. Quantifying the spatial variability of soil lead and developing a plan of action to reduce human exposure must be supported by the community for transitions to be successful. Choosing management options that the community supports is vital to the long-term sustainability of the project. If urban soil is deemed suitable for human activity and the desired land use falls in the “contact” category, the management preference of the community becomes an important component in the proposed framework.

For example, let us assume a community has tested their soil and the total lead value is 350 ppm lead. While that is below the USEPA guideline for residential soils, it is above the USEPA 2013 Technical Review Workgroup Lead Committee’s recommendation of 100 ppm for urban gardens. If the community desires a garden, there are several management options. The community may decide to embrace current best management practices. For urban gardens this usually includes raised-bed gardens. However, while some communities will have the resources to construct raised-bed garden, some may not. In addition to resource limitations, some communities may resist the notion that their soil is contaminated, and may desire instead to invest in their soil systems through amendments aimed at mitigating risk from soil lead. For example, during the construction of raised-bed gardens, a barrier is typically placed between existing soil systems and new soil that is imported. Communities that have been burdened by environmental pollutants and/or labeled “contaminated” or “toxic” in the past may resist the notion that they must cover up their soil and start anew [53]. Instead, improving existing soil systems may serve as a metaphor for communities desiring to invest in place, improving current conditions, setting the stage for a more sustainable future. Decisions made by the community can be influenced by attitudes and perceptions, cost, knowledge of soil testing and interpretation of results, legislative and governance obstacles, and physical land restrictions. Communities have the ultimate say in how they would like their soil systems managed; however, if communities choose an alternative management option that is not recommended from a public health perspective, the framework would suggest considering an alternative land use.

In what appears to be the case in many urban areas, the ubiquitous contamination of lead, including residential areas, can be daunting from a management perspective especially given the highly heterogeneous nature of contamination, the complexity of risk of exposure, and social and economic barriers to remediation. Perhaps one of the largest hurdles is the spatial extent of the contamination and in close proximity to where people live in urban areas, *i.e.*, residential land use (Figure 3) [2,32]. It is costly to excavate contaminated soil and find a safe place to dispose of the material. Moreover, soils form the brown infrastructure of cities and are too precious a resource to accept Clair Patterson’s prediction that “sometime in the near future it probably will be shown that the older urban areas of the United States have been rendered more or less uninhabitable by the millions of tons of poisonous industrial lead residues that have accumulated in cities during the past century” [54].

There is a growing desire to reclaim and use urban soil systems in a way that supports vibrant and sustainable cities [55]. Unfortunately, those trying to realize this desire are faced with conflicting guidelines that cause confusion and frustration. Differing guidelines for various activities can address risk exposure associated with those activities. For example, the USEPA recommendations for urban gardening (100 ppm) are tailored to specific activities. However, conflicting federal and state guidelines that address soil lead levels signal to communities that there is not a consensus among the scientific community regarding safe levels. For example, the California Office of Environmental Health Hazard Assessment recommends exercising caution when encountering soils that exceed 80 ppm. For older industrial cities, including Baltimore, this would mean exercising caution quite frequently, as 55%–77% of the soil sampled is elevated beyond 80 ppm (Table 1). Continuing to lower soil lead thresholds may in fact be more protective of human health but provides very little guidance to those who are actually working to improve urban soil conditions in a landscape that is widely contaminated. The framework provided here can help guide the emerging conversation regarding how to manage for both public health and sustainable vibrant cities.

## 5. Conclusions

The synthesis of multiple soil lead studies from the Baltimore Ecosystem Study that span spatial scales demonstrates that: (1) soil lead concentrations often exceed state and federal regulatory limits; (2) in residential areas, the variability of soil lead concentrations is often high; and (3) despite multiple sources and the highly heterogeneous and patchy nature of soil lead, discernable patterns do exist. This information is used to form a general framework for management, one that can support communities as they decide how to manage for soil lead and desired ecosystem services. The usefulness of the framework is dependent on clear, consistent, and protective soil lead guidelines (both general and activity specific) and support for rapid and reliable soil testing methodologies and best management practices are needed. Improving guidelines is a first step to achieving improved guidance that supports the shift to sustainable, working, urban soil systems.
